# 

**Published:** 1891-11

**Authors:** 


### Communications.

AMERICAN ACADEMY OF DENTAL SCIENCE.—At the 24th annual meeting and banquet of the American Academy of Dental Science, held at the Copley Square Hotel, Boston, Nov. 11th, 1891, the following officers were elected for the ensuing year: President, Dr. C. A. Brackett, of Newport; Vice-President, Dr. E. H. Smith, of Boston; Recording Secretary, Dr. C. H. Taft, of Cambridge; Corresponding Secretary, Dr. E. N. Harris, of Boston; Treasurer, Dr. V. C. Pond, of Boston, Librarian, Dr. E. C. Briggs, of Boston; Editor, Dr. Wm. H. Potter, of Boston; Executive Committee, Drs. F. E. Banfield, Boston; F. N. Seabury, Providence; Thos. Fillebrown, Boston.

CHAS. H. TAFT, *Rec. Sec'y.*

15 Brattle Square, Cambridge, Mass.

---

### MARYLAND STATE DENTAL ASSOCIATION.

The annual meeting of the Maryland State Dental Association will be held May 9, 10 and 11th 1892, instead of in December, 1891, as previously announced.

F. F. DREW, D. D. S.,
*Cor. Secretary.*

### To Readers and Correspondents.

Communications intended for publication, and exchange journals and papers, should be addressed to the Editor of The American Journal of Dental Science, No. 845 N. Eutaw St. Baltimore, Md. All letters relating to the business of the Journal, or containing cash remittances, to be directed to Messrs. Snowden & Cowman, No. 9 W. Fayette Street, Baltimore, Md. Articles for publication should be received by the Editor at least two weeks previous to the first of the month on which the number in which it is designed for them to appear, is issued.

☞ The American Journal of Dental Science will contain fifty pages of reading matter in each number, making a volume of about Six Hundred pages,

EXCLUSIVE OF ADVERTISEMENTS

